# H3.3-G34W in giant cell tumor of bone functionally aligns with the exon choice repressor hnRNPA1L2

**DOI:** 10.1038/s41417-024-00776-6

**Published:** 2024-05-29

**Authors:** Eunbi Lee, Yoon Jung Park, Anders M. Lindroth

**Affiliations:** 1 Graduate School of Cancer Science and Policy, Cancer Biomedical Science, National Cancer Center, Goyang-si, Republic of Korea; 2 Department of Nutritional Science and Food Management, Ewha Womans University, Seoul, Republic of Korea; 3 Graduate Program in System Health Science and Engineering, Ewha Womans University, Seoul, Republic of Korea

**Keywords:** Bone cancer, Functional genomics

## Abstract

RNA processing is an essential post-transcriptional phenomenon that provides the necessary complexity of transcript diversity prior to translation. Aberrations in this process could contribute to tumourigenesis, and we have previously reported increased splicing alterations in giant cell tumor of bone (GCTB), which carries mutations in the histone variant H3.3 encoding glycine 34 substituted for tryptophan (H3.3-G34W). G34W interacts with several splicing factors, most notably the trans-acting splicing factor hnRNPA1L2. To gain a deeper understanding of RNA processing in GCTB and isogenic HeLa cells with H3.3-G34W, we generated RNA-immunoprecipitation sequencing data from hnRNPA1L2 and H3.3-G34W associated RNAs, which showed that 80% overlapped across genic regions and were frequently annotated as E2F transcription factor binding sites. Splicing aberrations in both GCTB and HeLa cells with H3.3-G34W were significantly enriched for known hnRNPA1L2 binding motifs (*p* value < 0.01). This splicing aberration differed from hnRNPA1L2 knockouts, which showed alterations independent of H3.3-G34W. Of functional significance, hnRNPA1L2 was redistributed to closely match the H3.3 pattern, likely driven by G34W, and to loci not occupied in normal parental cells. Taken together, our data reveal a functional overlap between hnRNPA1L2 and H3.3-G34W with likely significant consequences for RNA processing during GCTB pathogenesis. This provides novel opportunities for therapeutic intervention in future modus operandi.

## Introduction

RNA processing is a crucial step in producing mature transcripts for protein translation. It has been estimated that almost all multiexon genes undergo alternative splicing, with approximately 100,000 alternative splicing events occurring in major human tissues [1]. To facilitate this intricate process, the spliceosome comprises five small nuclear RNAs and a set of over 100 associated splicing factors. Numerous studies have reported connections between cancer and splicing, providing detailed insight into the accumulation of aberrant splice variants [2]. In previous research, extensive RNA processing aberrations were described in giant cell tumor of bone (GCTB), which were linked to mutations in the histone variant H3.3 [3, 4]. GCTB is a benign tumor, and over 90% of patients carry a mutation of the histone variant H3.3, resulting in the substitution of glycine 34 with tryptophan (H3.3-G34W). Unlike many other cancers, GCTB display simple cytogenetics with no recurrent genetic alterations other than H3.3-G34W [5]. This provides an ideal situation for studies on the effects of gene expression and epigenetic alterations influenced by the mutation. The observed splicing aberrations almost exclusively affect exon skipping or inclusion. In GCTB tumors, exon inclusion (1254 events observed) was twice as common as exon skipping (661 events observed), suggesting that the stringency of exon choice is compromised by H3.3-G34W [4]. We identified hnRNPA1L2 as specifically interacting with H3.3-G34W, an auxiliary splicing component of a large family of trans-acting RNA-binding factors that has been associated with RNA processing [6]. The hnRNP family generally represses splicing in a sequence-dependent manner, although some members promote the process. The specific role of hnRNPA1L2 in exon choice is currently unknown.

Here we provide evidence using an isogenic and orthogonal model system of HeLa cells that the interaction between hnRNPA1L2 and H3.3-G34W aligns with the aberrant splicing and novel isoforms observed in GCTB. Immunoprecipitations of hnRNPA1L2-interacting RNA showed overlaps with genes involved in RNA processing and at E2F targeted genes, suggesting that splicing aberrations resulting from H3.3-G34W interacting with RNA processing may contribute to the tumorigenic process. The two proteins hnRNPA1L2 and H3.3-G34W co-localized on many exons, including genes with dense exon clusters.

In addition to altering RNA processing, GCTB carrying H3.3-G34W also transforms the epigenomic landscape, leading to a loss of DNA methylation and contributing to gene alterations in gene expression [4, 5]. This study highlights that oncohistones deviate from their normal histone counterparts and engage in aberrant activities that promote tumorigenesis by inducing excessive cell division. The relationship between chromatin and gene expression linked to RNA processing is an understudied area in tumorigenesis. However, research in simple cytogenetic backgrounds, such as that found in GCTB, promises to reveal essential information on chromatin-related RNA processing.

## Materials and methods

### Samples and cell lines

Using the zinc finger gene targeting technology previously described [4], we generated H3.3-WT and H3.3-G34W knock-in cell lines. The parental cells used were HeLa cells purchased from the Korean Cell Line Bank (https://cellbank.snu.ac.kr) which were authenticated with STR profiling. The cells were cultured in MEM, supplemented with 10% fetal bovine serum (FBS, #35-015-CV, Corning, USA) and 5 µg/ml gentamicin (#15710-064, Gibco, USA) under standard cell culturing conditions. RIP-seq analysis was performed using parental and two independent isogenic clones of each H3.3-WT and H3.3-G34W, while RNA-seq was performed using three each H3.3-WT and H3.3-G34W. This study used four H3.3-WT and eight H3.3-G34W samples of GCTB patient-derived primary cell lines, all obtained with patient consent approved by the IRB board of the National Cancer Center of Korea (IRB NCC2015-0070). The culture conditions were described in a previous report [4]. All cell lines in the study were regularly tested for Mycoplasma infection.

### CRISPR-mediated knockout of hnRNPA1L2

Short guide RNAs (sgRNA) were designed using the CRISPR-ERA algorithm (http://crispr-era.stanford.edu/) to target *hnRNPA1L2* and cloned into the SpCas9-2A-Puro plasmid pVX459 for CRISPR/Cas9 and sgRNA-mediated expression. The synthesized sgRNA sequence GCATGTCCTAAAGCTCTTTGagg was synthesized, cloned into the plasmid, transfected, and amplified in *E. coli* and then sequence-confirmed by Sanger sequencing. HeLa cell transfection was carried out using EndoFectin Max (#EF013, GeneCopoeia, USA). The cells were cultured in media supplemented with puromycin for 2–3 days, and then allowed to recover for 3 days. After that, the cells were FACS-sorted for individual clones in a 96-well format. The knockout clones were confirmed by RT-PCR and Western blot analysis.

### Library preparation for total RNA sequencing

For total RNA sequencing, three independent clones of HeLa cell lines were used, each with H3.3-WT, H3.3-G34W, and hnRNPA1L2 knockout in H3.3-WT (hnRNPA1L2-KO). Additionally, giant cell tumor of bone cell lines underwent RNA-seq analysis, with four being H3.3-WT and eight being H3.3-G34W. Total RNA was extracted using the RNeasy mini kit (#74104, Qiagen, Germany). For the sequencing analysis, we produced an RNA library using the Illumina TruSeq Stranded Total RNA LT Sample Prep Kit following the manufacturer’s protocol. We used a standard Illumina platform with 100 bp paired-end reads, generating over 100 million reads for each sample.

### RNA immunoprecipitation, RNA-seq extraction and analysis

Native RNA immunoprecipitation experiments were performed on HeLa parental cell line and two isoH3.3-G34W cell lines, as well as one isoH3.3-WT cell line, following the protocol described by Gagliardi and Matarazzo [7]. The cells were lysed using polysome lysis buffer (100 mM KCl, 5 mM MgCl_2_, 10 mM HEPES pH 7, 0.5% NP-40, 0.1 mM DTT, 20 U/ml ribonuclease Inhibitor RNaseOUT (#10777019, Invitrogen, USA), and EDTA-free Protease inhibitor cocktail (#04-693-159-001, Roche, Germany). Lysates were incubated with either GFP-Trap Agarose beads (#gta-20, Chromotek, Germany) or hnRNPA1L2 antibody (#ab180124, Abcam, UK) at +4 °C for 2 h and overnight at 4 °C, respectively. The lysates were then immobilized to ChIP-grade protein A/G magnetic beads (#26162, Thermo Scientific, USA). Antibody information is available in Supplementary Fig. 1c. After incubation, the beads were treated with proteinase K and RNA was isolated using standard phenol-chloroform extraction and ethanol precipitation. Library preparation for the RIP-seq samples was performed using TruSeq Stranded Total RNA with Ribo-Zero H/M/R_Gold for ribosomal RNA reduction. For total RNA samples, library preparation was performed using the SMARTer Ultra low input RNA library (PolyA+). The experiment was conducted on three isoH3.3-WT, three isoH3.3-G34W, and three hnRNPA1L2 knockout clones of isoH3.3-WT. Each RNA library was sequenced on an Illumina HiSeq2500 platform, which is capable of generating 100 bp paired-end reads. Between 6.5 and 9 million reads were generated for each library.

### Total RNA sequencing analysis

The raw reads were aligned to the reference human genome hg19 from UCSC (University of California Santa Cruz) with a reference gene annotation file using the STAR aligner (v2.7.10b) [8]. The reads in each sample were counted using FeatureCounts (v2.0.6) [9], and the differentially expressed genes between groups were calculated using the DESeq2 R package (v3.17) [10]. Statistically significant regulated genes were defined as those with an adjusted *p* value < 0.05 and |log_2_Foldchange| > 2.

For the analysis of differential alternative splicing analysis, rMATS-turbo (v4.1.2) and spliceR (v1.2.0) were employed [11, 12]. The outputs of rMATS were further analyzed with rMAPS (https://rnaseq-mats.sourceforge.net/) to identify RNA-binding proteins (RBPs) binding site around the rMATS exon-skipping events. The outputs of rMATS were also used in MASER (https://github.com/DiogoVeiga/maser/tree/master) to plot the splicing events as indexed events. Cuffdiff, which is part of the Cufflinks package (http://cole-trapnell-lab.github.io/cufflinks/) was employed to quantify changes in splice variant.

### RIP-sequencing analysis

The FASTQ files obtained from RIP sequencing were aligned to the human reference genome hg19, derived from UCSC, along with a reference gene annotation file using STAR (v2.7.10b). Peak calling was performed using MACS2 (v2.2.7.1) with FDR < 0.0001 [13]. The identified peaks were matched to Ensembl annotations for further analysis using ChIPpeakAnno (v3.1) [14]. Overlapping peaks between H3.3-G34W and hnRNPA1L2 were identified using the *findOverlapsOfPeaks* function from ChIPpeakAnno. Pathway analysis was conducted using the Enrichr online tool (https://maayanlab.cloud/Enrichr) on overlapping peaks. The distribution of peaks was identified using the Guitar R package. Exon numbers in all genes were tallied using the Guide to the Human Genome (Cold Spring Harbor Lab) and UCSC hg19 table browser tool.

## Results

### An orthogonal cell line with H3.3-G34W provide means for deep hnRNPA1L2 analysis

**Fig. 1:**
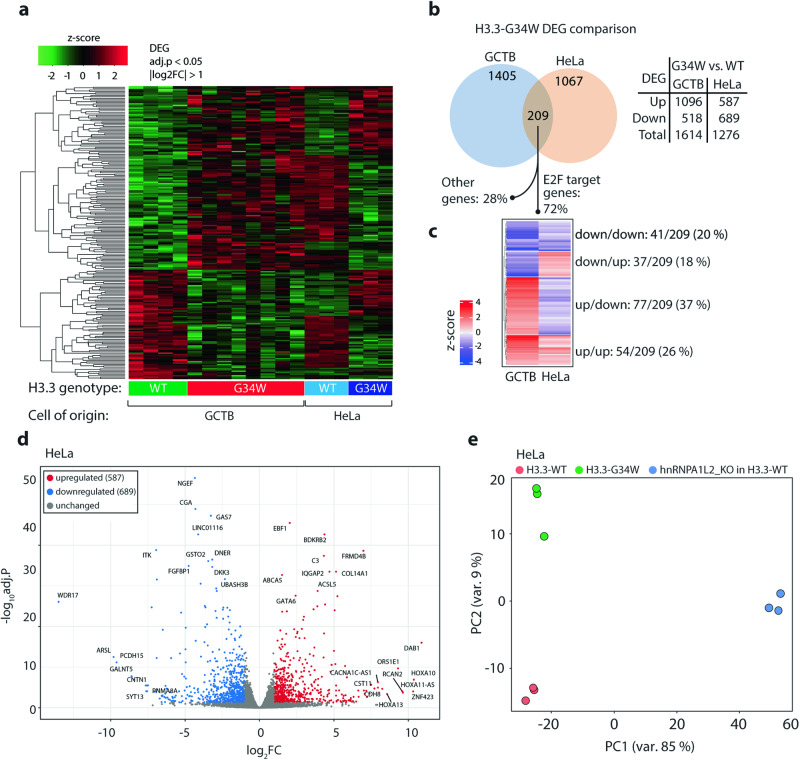
Gene expression analysis by RNA-seq of GCTB vs. HeLa cells harboring H3.3-G34W. **a** The results of hierarchical K-means clustering indicate distinct variations in DEGs. The heatmap depicts 12 GCTB (4 H3.3-WT and 8 -G34W) and 6 isogenic HeLa RNA-seq clones (three independent isogenic H3.3-WT and -G34W clones). **b** A comparison of WT vs. G34W in GCTB and HeLa extracted 209 overlapping DEGs of which 72% are E2F target genes. The total number of DEGs (adj. *p* < 0.05, |log_2_FC| > 1) is tabulated. **c** Hierarchical K-means clustering separates DEGs based on H3.3-G34W in GCTB and HeLa cells. The up- and down-regulated genes indicate cellular specificity of frequent E2F targets. **d** The volcano plot shows that there are 22% more down-regulated genes than up-regulated genes. **e** Multidimensional scaling was used to display the degree of separation between HeLa clones with WT and G34W genetic backgrounds, using a variance stabilizing transformation and showing the first two components. The knockout clones of hnRNPA1L2 cluster separately from either WT or G34W.

To analyze the intersection between hnRNPA1L2 and H3.3-G34W, we used the previously generated clones of HeLa cells that were gene-targeted to the H3F3A locus with a GFP-tagged H3.3-G34W (denoted isoH3.3-G34W). Parental HeLa cells or wild type isoH3.3-WT served as controls. Additionally, we used our panel of giant cell tumor of bone (GCTB) primary samples with their endogenous WT or G34W of H3.3. RNA-sequencing data from individual clones distinctly separated WT and G34W samples (Fig. 1a, c.f. Supplementary Fig. S1a). To determine the effect of H3.3-G34W on gene expression, we conducted hierarchical clustering of differentially expressed genes (DEG) by comparing GCTB stromal cells of mesenchymal origin with HeLa cells of epithelial origin. The results showed that approximately half of the DEGs shared between HeLa and GCTB had a similar expression pattern (Fig. 1a). The observed dichotomy is likely due to differences in the cells of origin, and H3.3 appears to contribute to DEGs depending on the cell type. A total of 1614 DEGs were statistically significant for GCTB, and 1276 for HeLa, with 209 genes shared between the two (see Fig. 1b). As per our previous study, we found that the DEGs in GCTB were targeted by the E2F transcription factor family [4]. Based on this, we estimate that 150 out of the 209 shared genes (72%) were targeted by E2F. The list of E2F target genes is available at the Broad Institute: Human Gene Set: HALLMARK_E2F_TARGETS and Encode. The E2F family of transcription factors (TFs) has been implicated in cancer due to its association with cell cycle regulation as a member of the CDK-RB-E2F nexus that promotes cell cycle entry through an E2F transcriptional program [15]. Hierarchical clustering indicated that the 209 genes targeted by E2F included both up-and down-regulated genes with approximately half (46%) being regulated similarly between GCTB and HeLa, and the other half (54%) showing an opposite pattern between the two cell types (Fig. 1c). A volcano plot indicates that isoH3.3-G34W results in both upregulated and downregulated genes (Fig. 1d). This suggests that H3.3-G34W governs a gene expression program of over one thousand genes, and that the overlap between the two diverse cell types converges to a substantial degree over E2F transcription factor regulation.

### Knockout of hnRNPA1L2 is distinct from its functional role in H3.3-G34W

To investigate the role of hnRNPA1L2 and its link to H3.3-G34W, hnRNPA1L2 was knocked out using CRISPR/Cas9-mediated gene targeting in isoH3.3-WT (Supplementary Fig. S1b). RNA-sequencing analysis was performed on the knockout (designated hnRNPA1L2-KO) in isoH3.3-WT and compared to non-knockouts in its parental isoH3.3-WT and -G34W. The three samples were distinctly separated, as judged from principal component analysis (Fig. 1e). The results suggest that the G34W follows a distinct trajectory from the loss of function of hnRNPA1L2. However, there may be a partial overlap, which we tested in subsequent experiments.

**Fig. 2:**
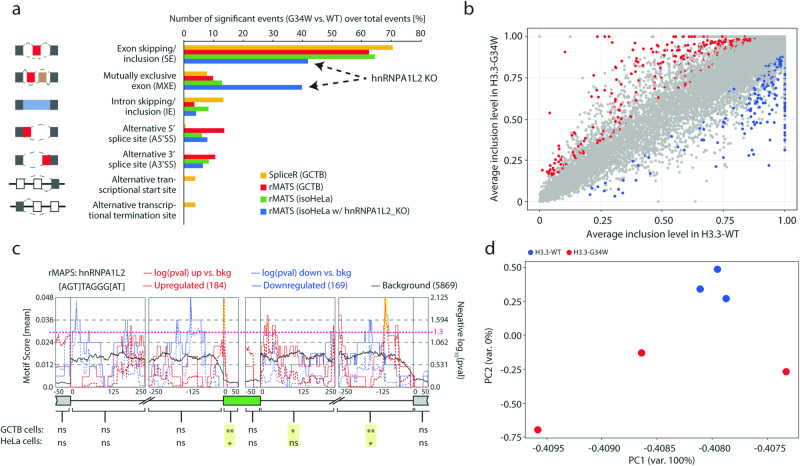
Splicing events in GCTB recapitulated in HeLa with H3.3-G34W vs. WT. **a** SpliceR and rMATS analyses reveal that exon skipping/inclusion (SE) is the most common significant event. Note that hnRNPA1L2 knockout clones show a drop in SE events (arrow) and an increase in mutually exclusive events (MXE). **b** The plot shows significant splicing events that satisfied both FDR < 0.05 and |ΔPSI| > 0.1, where ΔPSI (percent spliced-in) is calculated by subtracting the average inclusion level of WT from the average inclusion level of G34W based on triplicate samples. Gray dots represent non-significant events, while red and blue dots represent significant events. **c** The rMAPS plot, based on rMATS output, identifies hnRNPA1L2 motifs as a significant motif at upregulated SE events (red) in the intron/exon junction of the target exon and the 3’end of the downstream intron in both GCTB and isogenic HeLa cells. The smallest *p* value is indicated with a yellow box (*p < 0.05, **p < 0.01), and a dashed line at 1.3 indicates a *p* value of 0.05 with significant peaks marked yellow. **d** A principal component analysis plot of HeLa cells was created using MASER based on the rMATS output of significant SE events. The plot successfully separates WT from G34W, which also display clonal heterogeneity.

The hnRNPA1L2 protein is an RNA-binding auxiliary factor that works with other hnRNPs to perform various functions, mainly by binding exons as an exon choice suppressor [6]. To compare isogenic HeLa cells with GCTB harboring G34W [11, 12], we performed RNA-seq and splicing analysis using the rMATS and SpliceR computational tools. The analysis of RNA-seq data from H3.3-G34W-containing GCTB and HeLa cells using rMATS revealed that exon skipping and inclusion (SE) were the most common splicing events when compared to WT. These events accounted for 60–70% of the total events (Fig. 2a). Interestingly, knockout clones of hnRNPA1L2 in isoH3.3-WT cells resulted in fewer SE events and instead showed more pronounced mutually exclusive exon (MXE) events (as indicated by the arrows in Fig. 2a). This suggests that the SE events observed in H3.3-G34W align with hnRNPA1L2 without altering its function. As shown in Fig. 2b, isoH3.3-G34W exhibited a higher frequency of SE events compared to the control group. This may be due to a redistribution of hnRNPA1L2 in H3.3-G34W samples, resulting in an increase in the number of SE events. Given our previous findings of global epigenetic alterations in GCTB [5], we further investigated whether these increased SE events were affecting epigenetic modifiers. It seems that certain epigenetic modulators and DNA modifiers were significantly altered, as shown in Supplementary Fig. S2. Similar to our previous analysis of H3.3-G34W in GCTB, isoH3.3-G34W also displayed significant changes in splicing patterns. These changes may reflect the broad impact of hnRNPA1L2, which includes essential gene regulators and chromatin remodelers.

### Evidence of a significant overlap between hnRNPA1L2 and H3.3-G34W at target sites

We investigated whether the SE events in H3.3-G34W cells had hnRNPA1L2 binding motifs. To do this, we performed rMAPS analysis of the rMATS output to generate RNA splicing maps of RNA-binding proteins with defined binding motifs [16]. The hnRNPA1L2 protein binding to the canonical motif [AGT]TAGGG[AT], exhibited significant binding activity at the junction of target exons and downstream introns in both the GCTB and the HeLa transcriptomes. This finding further supports the association between H3.3-G34W and hnRNPA1L2, suggesting a potentially overlapping functional trajectory (see Fig. 2c). Out of the 115 RNA-binding proteins in the rMAPS test, hnRNPA1L2 was the third most significant RBP with a binding motif at and around the target exon (smallest *p* value in target exon 0.00784). The rMATS output in a multidimensional scaling plot also showed that H3.3-G34W clones dispersed while the H3.3-WT remained clustered (see Fig. 2d), suggesting that isoH3.3-G34W influenced exon target choice.

The analysis of splicing in both GCTB and isogenic HeLa with H3.3-G34W focuses on SE events. In both cases, there is significant evidence of hnRNPA1L2 binding in and around the target exon. Knockout of hnRNPA1L2 reduces SE events and favors mutually exclusive exon events.

### RNA immunoprecipitation indicates extensive overlap between hnRNPA1L2 and H3.3-G34W

**Fig. 3:**
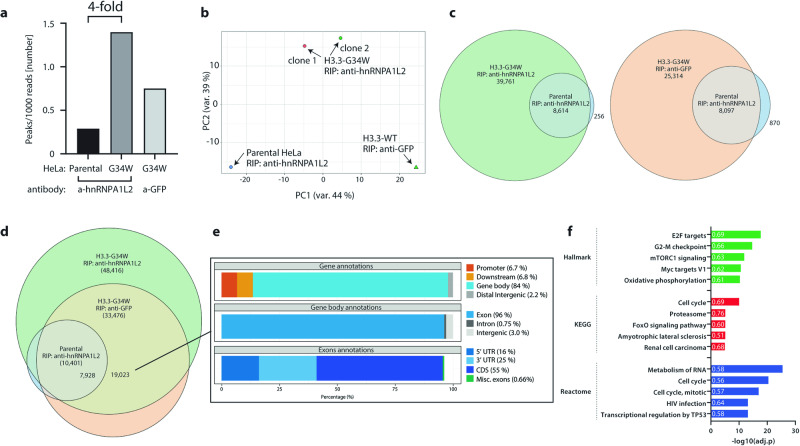
Close correlation of RIP-seq peaks of isoH3.3-G34W and hnRNPA1L2 in HeLa. **a** The tally of peak numbers over the number of reads after RIP-seq with anti-hnRNPA1L2 antibody shows a four-fold increase in hnRNPA1L2 peak numbers in the presence of H3.3-G34W. The GFP-tagged isoH3.3-G34W in the third bar serves as a control. **b** Multidimensional scaling of RIP-seq peaks separated by genetic background. **c** Venn diagrams indicate strong overlap of parental hnRNPA1L2 RIP-seq peaks with both GFP and hnRNPA1L2 IPs in isoH3.3-G34W clones pointing to their similar targeting regimens. **d** The Venn diagram displays the overlap between H3.3-G34W and hnRNPA1L2 on the genome, as indicated by RIP-seq peaks in isogenic H3.3-G34W HeLa with IP of hnRNPA1L2 and GFP-tagged H3.3-G34W. **e** The annotated regions from the 19,023 RIP-seq peaks from H3.3-G34W indicate that peak location is primarily at gene bodies and exons. **f** The GO-term analysis of RIP-peaks aligns with RNA-seq around E2F target genes, cell cycle control, and RNA metabolism. The numbers indicate fraction of genes identified in each term.

To determine the extent of overlap between hnRNPA1L2 and H3.3-G34W during transcription and RNA processing, we performed native, non-crosslinked RIP-seq [7] to immunoprecipitate and sequence hnRNPA1L2-associated RNA from isoH3.3-G34W and control cells. Our main objective was to identify the target RNA and broadly infer a link to the RNAs associated with H3.3-G34W. In this context, the ratio of hnRNPA1L2-peaks to the total number of reads was approximately four times higher in isoH3.3-G34W than in the parental control. This suggest that hnRNPA1L2 has novel G34W-assigned targets (Fig. 3a). In contrast, the RIP-peaks from GFP-tagged H3.3-G34W are less pronounced (rightmost bar in Fig. 3a). In accordance with the observed increase in SE events and the presence of distinct hnRNPA1L2 motifs, the hnRNPA1L2-peaks from the parental control strongly overlapped with both isoH3.3-G34W and its hnRNPA1L2-precipitates (see Fig. 3c). It is worth noting that isoH3.3-G34W (anti-GFP) precipitates a significantly larger number of transcripts than hnRNPA1L2 alone, but when used together, this number increases considerably (see Fig. 3c). The overlap between isoH3.3-G34W RIP-peaks and parental HeLa amounted to 80%, with a noticeable increase in isoH3.3-G34W peaks (Fig. 3d). Of the 19,023 H3.3-G34W peaks that overlapped with hnRNPA1L2-peaks at annotated genomic areas, 84% were found at gene bodies and only 2.2% at distal intergenic areas. Almost all of these peaks (96%) were over exons (Fig. 3e). In contrast to hnRNPA1L2 motifs, which appear to occur in both introns and exons, the estimate for their occurrence has not been quantified and may primarily be at exons (see Figs. 2c and 3e). We investigated whether there is an overlap between genes with SE events and genes with RIP-seq peaks. We found that 952 genes in isoH3.3-G34W had splicing events, and of those, 432 genes had RIP-seq peaks, which accounts for approximately 45% of aberrantly spliced genes with SE events. The estimated equivalent for GCTB was 42%, suggesting functional overlap between hnRNPA1L2 and H3.3-G34W.

The analysis of GO-terms for the 19,023 RIP-peaks once again indicated an association with E2F target genes and cell cycle regulation. This was previously suggested among the 209 overlapping DEGs from the RNA-seq analysis comparing HeLa and GCTB (refer to Figs. 1c and 3f). The close association of genomic targets between hnRNPA1L2 and isoH3.3-G34W, both intragenic and centered around E2F targets, suggests that the two proteins share a common trajectory and may contribute to tumorigenesis by dysregulating E2F function.

### hnRNPA1L2 functionally aligns with H3.3-G34W at known and novel genes

**Fig. 4:**
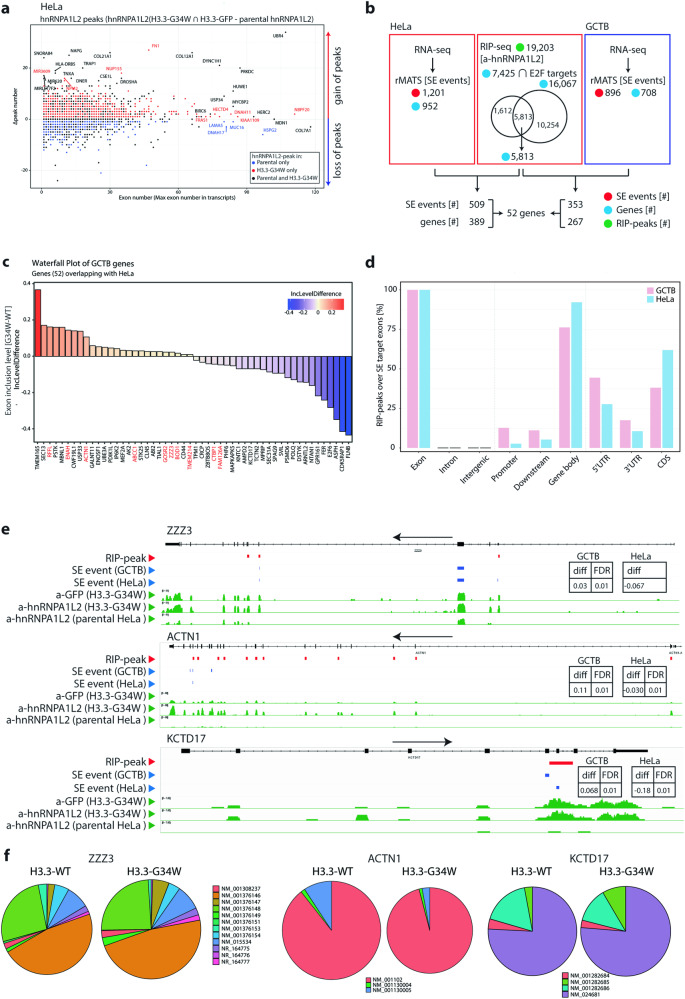
Gene-by-gene analysis of hnRNPA1L2 RIP-seq peaks. **a** The scatter plot displays the delta hnRNPA1L2 peak numbers of H3.3-G34W minus parental. The plot indicates peaks only detected in parental (blue dots), peaks only in H3.3-G34W (red dots), and peaks in genes of both parental and H3.3-G34W (black dots). For example, *NBPF20* is a novel target that appears only in H3.3-G34W. **b** A comparison between splicing events from RNA-seq and RIP-seq events in H3.3-G34W in HeLa (red squares) and GCTB (blue square) over E2F targets. The 52 common genes were identified by matching the extracted SE events to annotated genes. **c** Waterfall plots show the inclusion level difference of these genes in GCTB. Ten genes in GCTB overlapped between RIP-seq peaks and SE events (i.e., coordinates over exons) and are marked in red text, while the remaining genes did not overlap over the same exons. **d** Of the 52 genes that showed perfect overlap between hnRNPA1L2 and SE events in both GCTB and HeLa, they were enriched predominantly over gene bodies. **e** Candidate genes from the 52 gene list showing overlap between SE events and RIP peaks. IGV traces of *ZZ3*, *ACTN1*, and *KCTD17* show delta SE events of 0.03 in GCTB and −0.1 in HeLa, both at the false discovery rate of 0.01. **f** The pie-charts illustrate changes in isoforms in GCTB. Note that the parental does not have hnRNPA1L2 in these candidates.

In a previous study of isogenic HEK293 cells, hnRNPA1L2 was identified as a novel interacting partner to G34W after immunoprecipitation followed by mass spectrometry [4]. The significant overlap between hnRNPA1L2 and H3.3-G34W (Fig. 3a, c), the increased SE events that significantly carry hnRNPA1L2 motifs (Fig. 2a, c), and the redistribution of events in hnRNPA1L2-KO (Fig. 2a), suggests that the two molecules intersect and may share functional avenues related to GCTB tumorigenesis. The study aimed to determine if RIP-seq peaks were directed toward novel or known G34W targets. In this study, it was discovered that transcripts in the isoH3.3-G34W samples were more frequently associated with hnRNPA1L2 compared to parental cells. This was exemplified by the E3 ubiquitin ligase *UBR4*, which increased 18-fold (Fig. 4a and Supplementary Fig. 3a, b). Additionally, novel transcripts not targeted in parental cells, such as the E3 ubiquitin ligase *HECTD4* were identified. However, some transcripts lost hnRNPA1L2 association in isoH3.3-G34W. It was hypothesized that genes with a high exon count would be more frequently affected, but an analysis of the correlation coefficient *R* analysis did not support this assumption (see Supplementary Fig. 3). The number of RIP-peaks unique to H3.3-G34W as seen in Fig. 4a was three times higher than in the parental group (red markers: 4094 genes, blue markers: 1281 genes), while the proportion of SE events in those genes was similar (5.8% vs. 6.3%, respectively). This indicates that more peaks lead to more events, indicating that hnRNPA1L2 is directed to new targets via its association with H3.3-G34W.

### An E2F nexus of cell cycle control emanates among candidate genes

To identify candidate genes targeted by hnRNPA1L2 in the H3.3-G34W background, we extracted E2F transcription factor targeted genes with RIP peaks that also had skipped exon (SE) events overlapping between HeLa and GCTB. We started with the SE events and hnRNPA1L2 peaks in HeLa, resulting in 389 genes to compare with 267 genes in GCTB. Together, they fulfilled the criteria of having hnRNPA1L2 peaks, being targeted by E2F, and displaying SE events. This comparison resulted in 52 genes that overlapped between HeLa and GCTB (Fig. 4b). The waterfall plot in Fig. 4c displays the SE events of 52 genes as candidates at the nexus of hnRNPA1L2-H3.3-G34W-E2F. The list includes genes encoding actin-associated proteins that are essential in shaping and anchoring actin, building intracellular structures centered on CD44, and cell-cell interactions (e.g., *ACTN1*, *SVIL*, *TPM1* and *FLNB*). We also found genes encoding proteins linked to cytoskeleton remodeling (e.g., *ENAH*, *ABI2*, *MBLN1*, and *TIAL1*). The study identified genes related to ubiquitination and protein homeostasis, such as *PSMD6* and *UBE3A*, as well as genes involved in protein transport and components of the nuclear pore complex, such as *SEC31A*, *GOSR2*, and *SEC13*. Additionally, genes associated with chromatin, such as *ZZZ3*, *CTBP1*, and *EEF1D*, and transcription and translation regulators, such as *E2F6*, *PHF6*, and *MEF2A* were among the targets. These genes are diverse examples of genes with SE events where hnRNPA1L2 and H3.3-G34W coincide based on their interaction, strongly suggesting a functional association. SE events align mainly with exons over annotated gene features (Fig. 4d). We also found instances of direct overlap between RIP-seq peaks and SE events (Fig. 4e) that resulted in quantitative changes in splice variants (Fig. 4f).

**Fig. 5:**
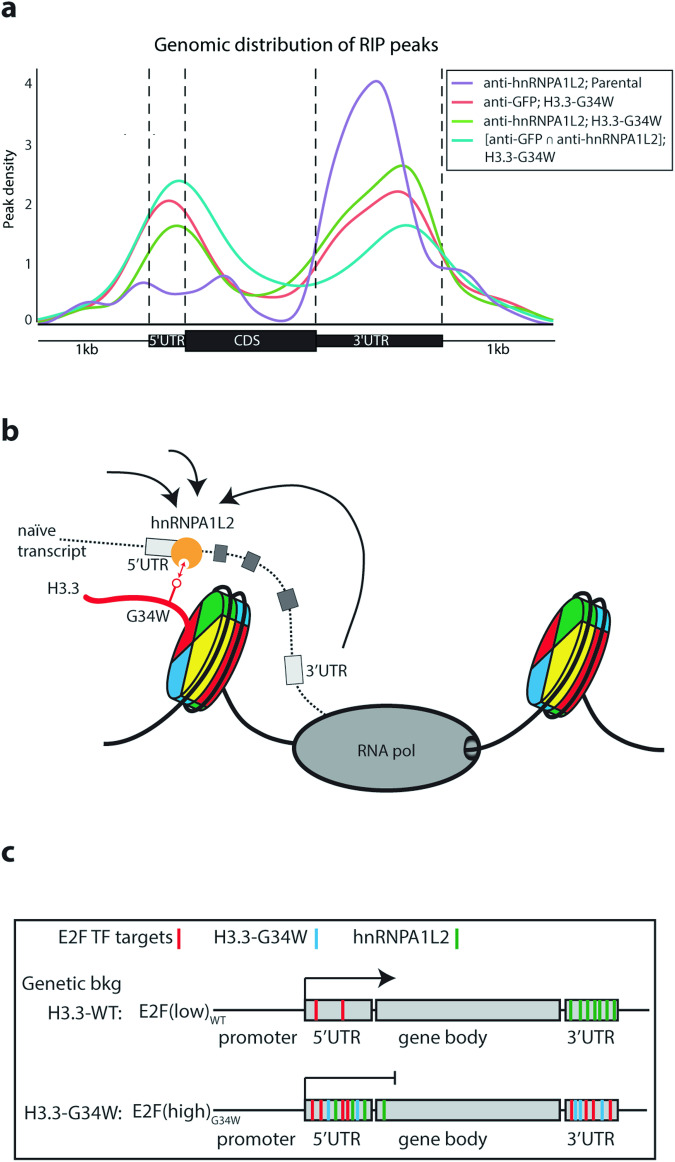
Genic distribution of hnRNPA1L2 uncovers H3.3-G34W mimicry. **a** The RIP-seq peak density of hnRNPA1L2 over annotated genic regions accumulates over the 3’UTR in WT (purple line), but partially redistributes over the 5’UTR in H3.3-G34W (green line). **b** hnRNPA1L2 targets exons for suppression and in the process becomes hijacked by H3.3-G34W to the histone H3.3 normal distribution locations. Importantly, the number of hnRNPA1L2 peaks increases 4-fold in H3.3-G34W. **c** This illustration shows the increased E2F targeting at the 5’UTR in H3.3-G34W by hnRNPA1L2 (represented by green bars), which contributes to reduced SE events at the 3’UTR and elevated promoter suppression at the 5’UTR through E2F suppression (such as E2F4 and 6).

To gain a better understanding of where they would exert their joint function on the gene body, the RIP-seq peaks were graphed. The results showed that hnRNPA1L2 in parental cells mainly associates with the 3’UTR, while H3.3-G34W were distributed over both 3’UTR and 5’UTR (Figs. 5a and 4e). Notably, hnRNPA1L2 relocated almost half of its peaks to the upper coding region in the H3.3-G34W genetic background, which was further evident when plotting overlapping peaks of H3.3-G34W and hnRNPA1L2. It is noteworthy that our analysis has assigned most hnRNPA1L2 peaks to the exons which include 5’-exons (Fig. 3e). These results suggest a significant overlap between hnRNPA1L2 and H3.3 when altered to accommodate G34W.

## Discussion

Giant cell tumor of bone is a benign neoplasm that causes local bone destruction and stromal overgrowth, requiring intermittent curettage. The meta-epiphyseal regions of the long bones are the most frequently affected areas [17]. Bone destruction results from increased osteoclast activity, and reduced osteoblast bone formation, resulting in a net loss of bone. The stromal cell compartment of the tumor is quite heterogenous consisting of mesenchymal cells, monocytic cells, and multinucleated giant cells [18]. The cytokines macrophage colony-stimulating factor (M-CSF) and receptor activator of nuclear factor-κB (RANK) and its ligand RANKL drive the fusion of precursors of a monocytic lineage to form terminally differentiated multinucleated giant cells [19]. The stromal overgrowth originates from poorly defined mesenchymal cells that almost always carry a heterozygous G-to-T mutation of *H3F3A* ( > 90%, c.103G > T), which encodes H3.3-G34W [20, 21]. Recent evidence suggests that these mesenchymal cells are unable to differentiate and exhibit mesenchymal stem cell-like properties [5, 22]. The link between this feature and its proliferation, as well as the genes involved in this process is currently unknown and has not been experimentally tested.

In a previous study, we demonstrated an association between H3.3-G34W and hnRNPA1L2, indicating a connection between RNA processing (where hnRNPA1L2 exerts its function) and chromatin (where H3.3 is deposited). To this end, we introduced H3.3-G34W into the HeLa cell line of epithelial origin and compared its effects on gene expression and RNA processing. We did this in knowing that H3.3-G34W is heterozygous and the only known genetic alteration identified in GCTB, suggesting that it imposes dominant-negative features at locations where it is deposited [20, 21]. Although the intersection between chromatin and RNA processing has been suggested since the 1990s, direct evidence was not provided until 2009 by the labs of Ast and Guigó [23, 24]. They demonstrated that the mediation between chromatin and RNA processing involves the transcriptional process, recognition of intro/exon junctions via specific motifs, and the structure of chromatin and its epigenetic modifications. As regards to the interaction between the splicing factor hnRNPA1L2 and H3.3-G34W, we have here demonstrated their functional alignment through the following observations: (1) the splicing aberrations events in GCTB carried canonical hnRNPA1L2 binding motifs; (2) the RIP-seq peaks of hnRNPA1L2 in the H3.3-G34W genetic background increased four-fold and strongly overlapped with transcripts associated with H3.3-G34W; and (3) hnRNPA1L2 redistributed from the 3’UTR to follow the pattern of H3.3-G34W distribution over the 5’UTR. The function of the G34W-enforced hnRNPA1L2 redistribution differed from the observed splicing aberrations in hnRNPA1L2-KO samples. This resulted in reduced SE events and increased MXE events, which were distinct from H3.3-G34W. These findings suggest that G34W does not alter the function of hnRNPA1L2, but only its location. The study found that isogenic HeLa cells with H3.3-G34W were able to recreate the splicing aberrations observed in GCTB, providing further evidence of the strong influence on RNA processing by histone H3.3 in its altered state. If hnRNPA1L2 acts as an exon choice suppressor, its forced relocalization to the 5’UTR may lead to less exon suppression and more exon inclusion, as shown in Fig. 4a. The study emphasizes the significance of auxiliary splicing factors in association with chromatin. There are documented cases of association with the C-terminal domain of RNA polymerase II, which connects transcription, splicing, polyadenylation and cleavage [25].

This study demonstrates that the selection of exons for the mature transcript is determined by the chromatin while the transcripts are still attached to the RNA polymerase (Fig. 5). In cancer, this could result in cell cycle control being governed by RNA transcription and processing, potentially leading to significant consequences due to aberrations in exon choice (Fig. 5b). Future research will need to test if a redistribution of hnRNPA1L2 toward 5’UTR by H3.3-G34W potentiates altered RNA processing, in effect suppressing E2F target genes. Currently, the osteoclast-limiting RANKL inhibitor denosumab is widely used for therapeutic treatment of GCTB. According to our findings, personalized medicine strategies should investigate combinatorial drug interventions targeting hnRNPA1L2, possibly with the specific inhibitor quercetin as an extension of therapy in the H3.3-G34W genetic background [26]. Our data suggest that therapeutic interventions that break the association between H3.3-G34W and other auxiliary factors of the RNA processing machinery may be a successful avenue to explore in effective GCTB treatment.

### Supplementary information

Supplementary Table 1

Supplementary Fig. 1

Supplementary Fig. 2

Supplementary Fig. 3

## Data Availability

The datasets used or analyzed in this article are available upon request from the corresponding author.

## Code availability

The code for differential expression and splicing analyses can be found at https://github.com/Eunbi9561/hnRNPA1L2-paper.
