# Correction: H3.3-G34W in giant cell tumor of bone functionally aligns with the exon choice repressor hnRNPA1L2

**DOI:** 10.1038/s41417-024-00803-6

**Published:** 2024-06-27

**Authors:** Eunbi Lee, Yoon Jung Park, Anders M. Lindroth

**Affiliations:** 1 Graduate School of Cancer Science and Policy, Cancer Biomedical Science, National Cancer Center, Goyang-si, Republic of Korea; 2 Department of Nutritional Science and Food Management, Ewha Womans University, Seoul, Republic of Korea; 3 Graduate Program in System Health Science and Engineering, Ewha Womans University, Seoul, Republic of Korea

**Keywords:** Bone cancer, Functional genomics

Correction to: *Cancer Gene Therapy* 10.1038/s41417-024-00776-6, published online 29 May 2024

In the Acknowledgements section of this article, one additional grant number relating to the National Cancer Center of Korea was missing and should have been 2210530. The original article has been corrected.

