# Supplementary material for: H3.3-G34W in giant cell tumor of bone functionally aligns with the exon choice repressor hnRNPA1L2
Source: Cancer Gene Ther. 2024 May 29;31(8):1177–85. doi: 10.1038/s41417-024-00776-6 (PMC11327103; doi:10.1038/s41417-024-00776-6)
Supplement: Supplementary file 1 — Supplementary Table 1 [file 41417_2024_776_MOESM1_ESM.pdf]

Sample name	Cell line	Genotype	Knockout	Method	Antibody	Illumina library prep kit	Types of Read	Data number
GCTB_07	GCTB	H3.3-G34W	N/A	PolyA+ RNA-seq	N/A	TruSeq Stranded Total RNA LT Sample Prep Kit (Gold)	Paired-end	1603AHS-0003,1604KHP-0051
GCTB_08	GCTB	H3.3-WT	N/A	PolyA+ RNA-seq	N/A	TruSeq Stranded Total RNA LT Sample Prep Kit (Gold)	Paired-end	1603AHS-0003,1604KHP-0051
GCTB_09	GCTB	H3.3-WT	N/A	PolyA+ RNA-seq	N/A	TruSeq Stranded Total RNA LT Sample Prep Kit (Gold)	Paired-end	1603AHS-0003,1604KHP-0051
GCTB_10	GCTB	H3.3-G34W	N/A	PolyA+ RNA-seq	N/A	TruSeq Stranded Total RNA LT Sample Prep Kit (Gold)	Paired-end	1603AHS-0003,1604KHP-0051
GCTB_11	GCTB	H3.3-WT	N/A	PolyA+ RNA-seq	N/A	TruSeq Stranded Total RNA LT Sample Prep Kit (Gold)	Paired-end	1603AHS-0003,1604KHP-0051
GCTB_12	GCTB	H3.3-G34W	N/A	PolyA+ RNA-seq	N/A	TruSeq Stranded Total RNA LT Sample Prep Kit (Gold)	Paired-end	1603AHS-0003,1604KHP-0051
GCTB_13	GCTB	H3.3-G34W	N/A	PolyA+ RNA-seq	N/A	TruSeq Stranded Total RNA LT Sample Prep Kit (Gold)	Paired-end	1802AHP-0047
GCTB_14	GCTB	H3.3-WT	N/A	PolyA+ RNA-seq	N/A	TruSeq Stranded Total RNA LT Sample Prep Kit (Gold)	Paired-end	1802AHP-0047
GCTB_15	GCTB	H3.3-G34W	N/A	PolyA+ RNA-seq	N/A	TruSeq Stranded Total RNA LT Sample Prep Kit (Gold)	Paired-end	1802AHP-0047
GCTB_16	GCTB	H3.3-G34W	N/A	PolyA+ RNA-seq	N/A	TruSeq Stranded Total RNA LT Sample Prep Kit (Gold)	Paired-end	1802AHP-0047
GCTB_17	GCTB	H3.3-G34W	N/A	PolyA+ RNA-seq	N/A	TruSeq Stranded Total RNA LT Sample Prep Kit (Gold)	Paired-end	1802AHP-0047
GCTB_18	GCTB	H3.3-G34W	N/A	PolyA+ RNA-seq	N/A	TruSeq Stranded Total RNA LT Sample Prep Kit (Gold)	Paired-end	1802AHP-0047
HeLa2_WTA6	HeLa	H3.3-WT	N/A	PolyA+ RNA-seq	N/A	TruSeq Stranded Total RNA LT Sample Prep Kit (Human Mouse Rat)	Paired-end	1811KNO-0351
HeLa3_WTB2	HeLa	H3.3-WT	N/A	PolyA+ RNA-seq	N/A	TruSeq Stranded Total RNA LT Sample Prep Kit (Human Mouse Rat)	Paired-end	1811KNO-0351
HeLa5_WTC2	HeLa	H3.3-WT	N/A	PolyA+ RNA-seq	N/A	TruSeq Stranded Total RNA LT Sample Prep Kit (Human Mouse Rat)	Paired-end	1811KNO-0351
HeLa6_GWA4	HeLa	H3.3-G34W	N/A	PolyA+ RNA-seq	N/A	TruSeq Stranded Total RNA LT Sample Prep Kit (Human Mouse Rat)	Paired-end	1811KNO-0351
HeLa7_GWA6	HeLa	H3.3-G34W	N/A	PolyA+ RNA-seq	N/A	TruSeq Stranded Total RNA LT Sample Prep Kit (Human Mouse Rat)	Paired-end	1811KNO-0351
HeLa8_GWB6	HeLa	H3.3-G34W	N/A	PolyA+ RNA-seq	N/A	TruSeq Stranded Total RNA LT Sample Prep Kit (Human Mouse Rat)	Paired-end	1811KNO-0351
Parental_1	HeLa	Parental	hnRNPA1L2	PolyA+ RNA-seq	N/A	TruSeq Stranded mRNA LT Sample Prep Kit	Paired-end	HN00125921
Parental_17	HeLa	Parental	hnRNPA1L2	PolyA+ RNA-seq	N/A	TruSeq Stranded mRNA LT Sample Prep Kit	Paired-end	HN00125921
Parental_20	HeLa	Parental	hnRNPA1L2	PolyA+ RNA-seq	N/A	TruSeq Stranded mRNA LT Sample Prep Kit	Paired-end	HN00125921
WTB2_3	HeLa	H3.3-WT	hnRNPA1L2	PolyA+ RNA-seq	N/A	TruSeq Stranded mRNA LT Sample Prep Kit	Paired-end	HN00125921
WTB2_5	HeLa	H3.3-WT	hnRNPA1L2	PolyA+ RNA-seq	N/A	TruSeq Stranded mRNA LT Sample Prep Kit	Paired-end	HN00125921
WTC2_1	HeLa	H3.3-WT	hnRNPA1L2	PolyA+ RNA-seq	N/A	TruSeq Stranded mRNA LT Sample Prep Kit	Paired-end	HN00125921
GWA4_7	HeLa	H3.3-G34W	hnRNPA1L2	PolyA+ RNA-seq	N/A	TruSeq Stranded mRNA LT Sample Prep Kit	Paired-end	HN00125921
RIP-hnRNP_2	HeLa	Parental	N/A	RIP-seq	anti-hnRNPA1L2	TruSeq Stranded Total RNA with Ribo-Zero H/M/R_Gold for ribosomal RNA reduction	Paired-end	HN00127686
GW-B6-GFP_1	HeLa	H3.3-G34W	N/A	RIP-seq	anti-GFP	TruSeq Stranded Total RNA with Ribo-Zero H/M/R_Gold for ribosomal RNA reduction	Paired-end	HN00130164
GW-A6-hnRNP	HeLa	H3.3-G34W	N/A	RIP-seq	anti-hnRNPA1L2	TruSeq Stranded Total RNA with Ribo-Zero H/M/R_Gold for ribosomal RNA reduction	Paired-end	HN00178095
GW-B6-hnRNP	HeLa	H3.3-G34W	N/A	RIP-seq	anti-hnRNPA1L2	TruSeq Stranded Total RNA with Ribo-Zero H/M/R_Gold for ribosomal RNA reduction	Paired-end	HN00178095
WTB2_input	HeLa	H3.3-WT	N/A	Input	N/A	TruSeq DNA Sample prep Kit	Paired-end	HN00114580

GCTB tumor samples
HeLa RNA-seq
HeLa hnRNPA1L2 knockouts
HeLa RIP-seq
