# Supplementary figures and images for: H3.3-G34W in giant cell tumor of bone functionally aligns with the exon choice repressor hnRNPA1L2
Source: Cancer Gene Ther. 2024 May 29;31(8):1177–85. doi: 10.1038/s41417-024-00776-6 (PMC11327103; doi:10.1038/s41417-024-00776-6)

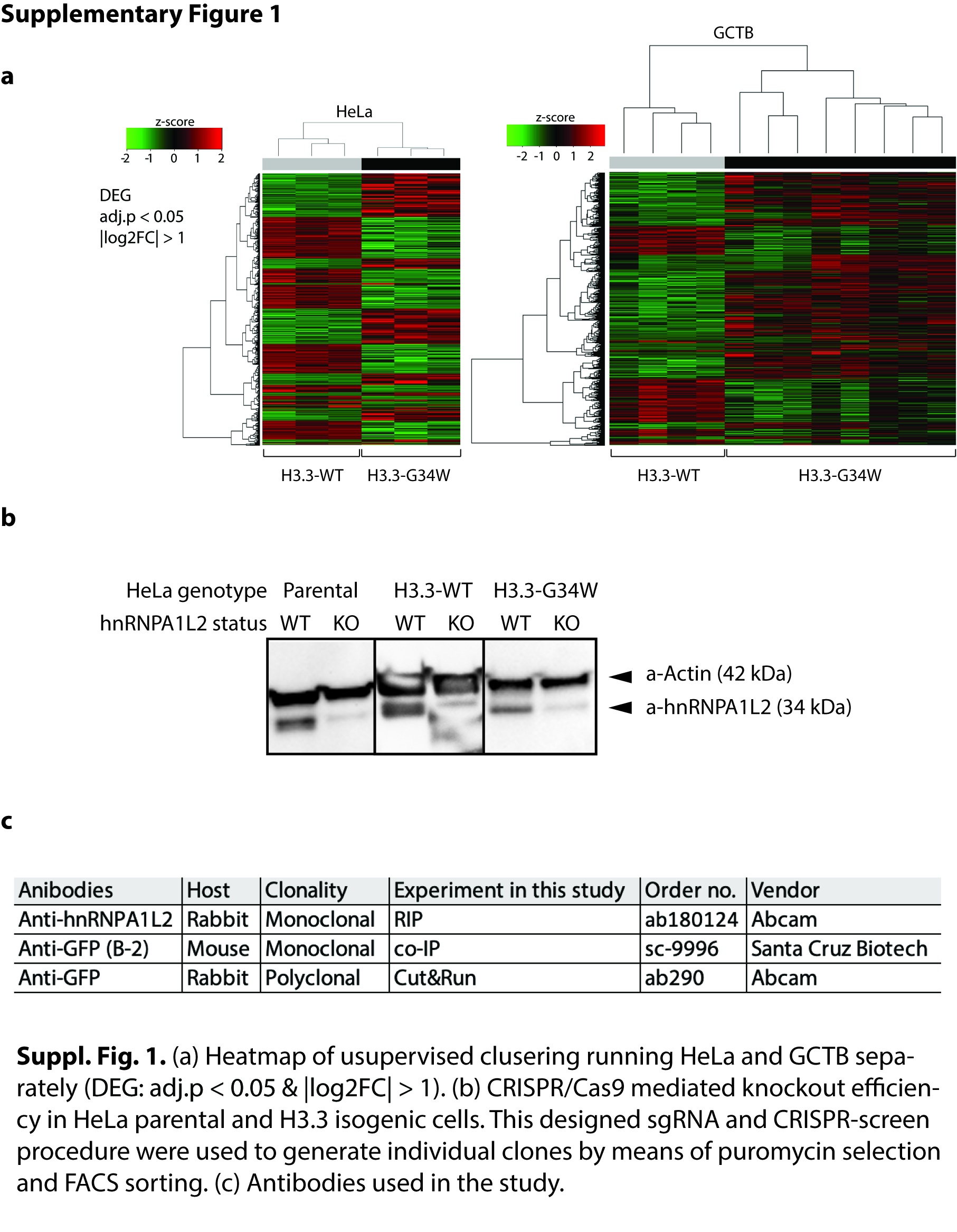

Supplement: Supplementary file 2 — Supplementary Fig. 1 [file 41417_2024_776_MOESM2_ESM.tif]

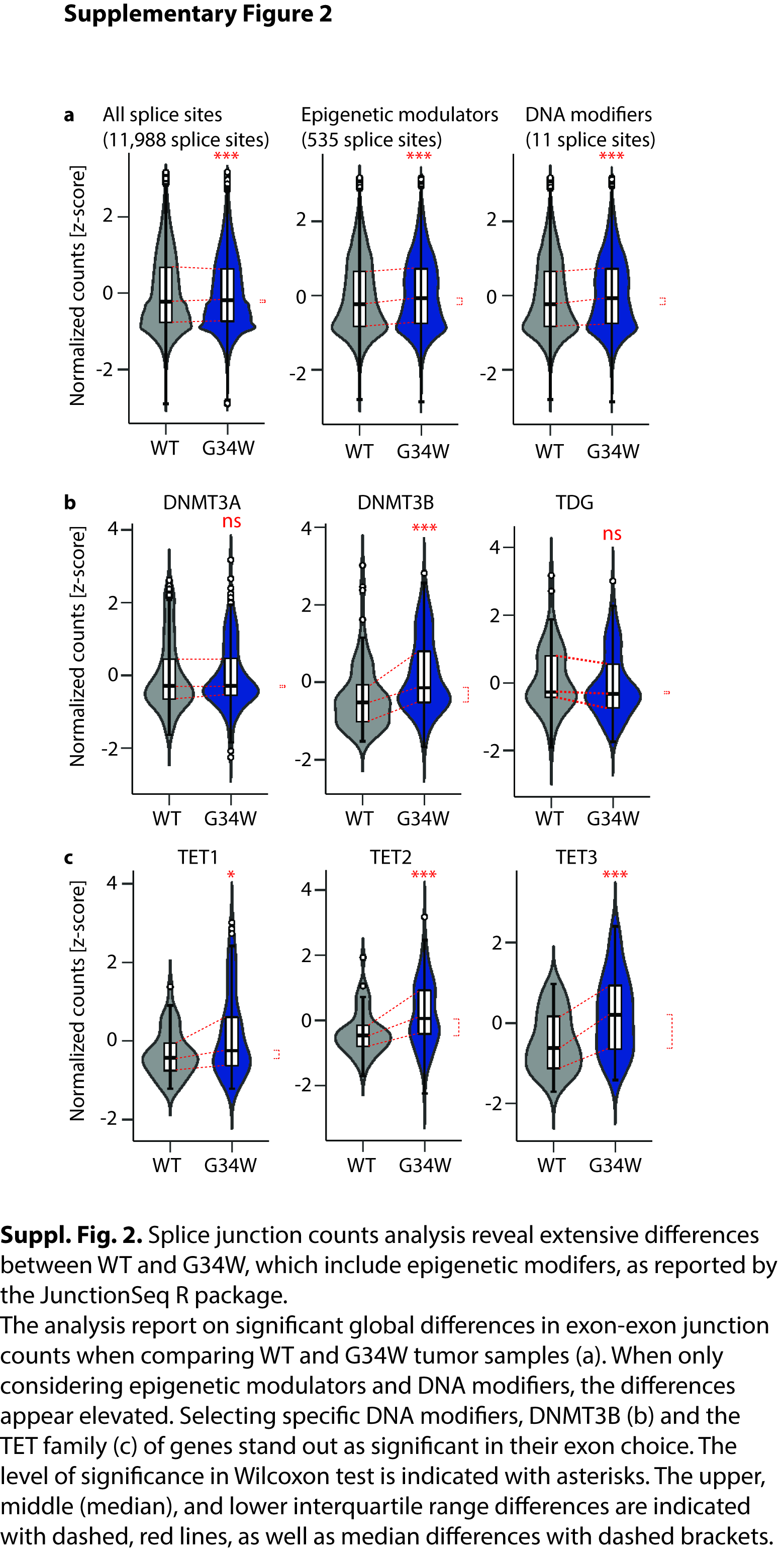

Supplement: Supplementary file 3 — Supplementary Fig. 2 [file 41417_2024_776_MOESM3_ESM.tif]

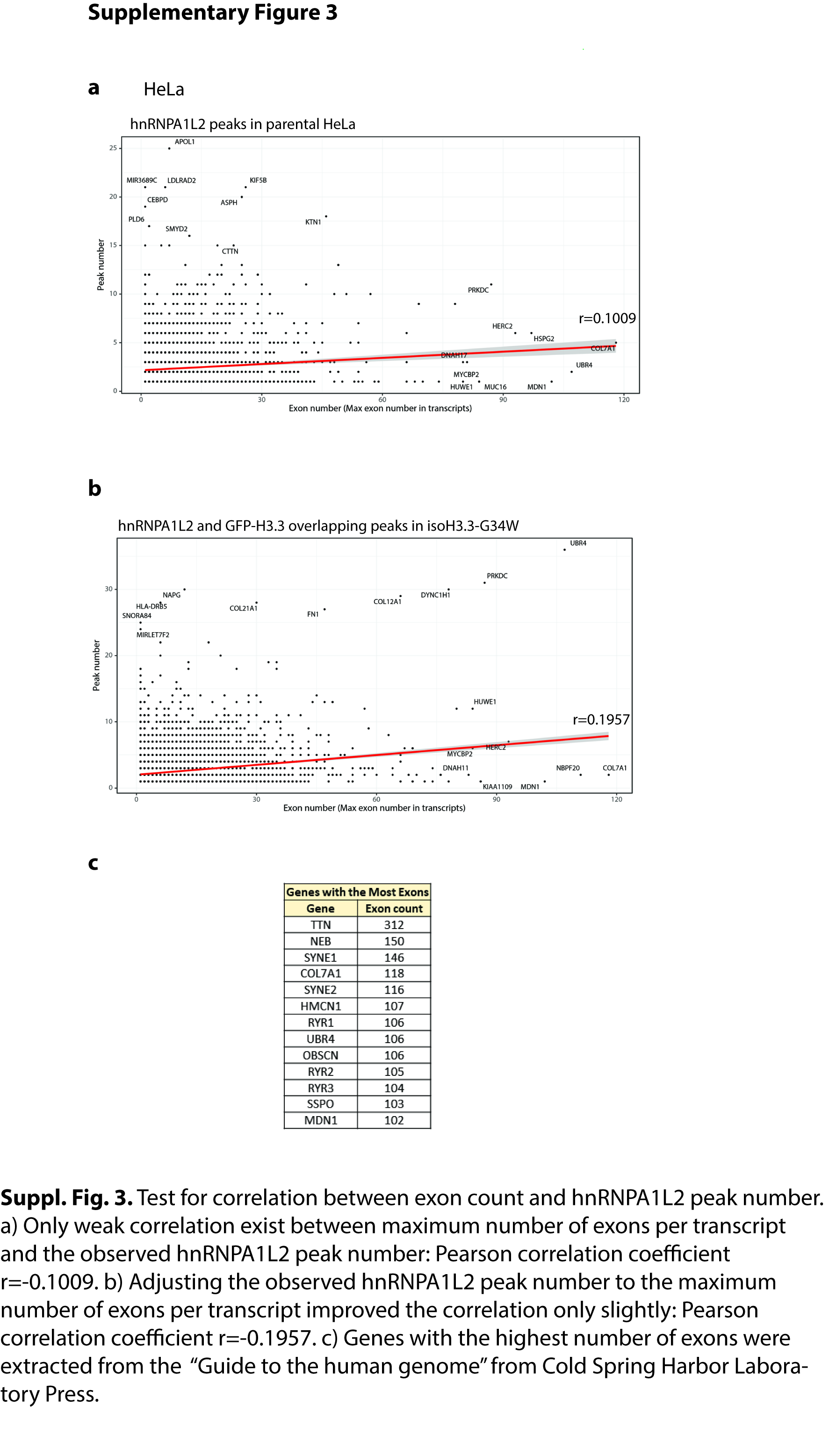

Supplement: Supplementary file 4 — Supplementary Fig. 3 [file 41417_2024_776_MOESM4_ESM.tif]
